# Gliosarcoma Invading the Temporal Bone, Temporalis Muscle, and Skull Base

**DOI:** 10.7759/cureus.42319

**Published:** 2023-07-23

**Authors:** Khalid T Alghamdi, Alaa A Ashqar, Ahmad Alamoudi, Abdullah Alzahrani, Alaa Samkari, Hussam Kutub

**Affiliations:** 1 Neurosurgery, King Faisal Specialist Hospital and Reseach Centre, Riyadh, SAU; 2 Medicine, King Saud Bin Abdulaziz University for Health Sciences, Jeddah, SAU; 3 Medicine, King Saud Bin Abdulaziz University for Health Sciences College of Medicine, Jeddah, SAU; 4 General Surgery, King Saud Bin Abdulaziz University for Health Sciences, Jeddah, SAU; 5 Pathology and Laboratory Medicine, King Abdulalziz Medical City, Jeddah, SAU; 6 Neurosurgery, King Abdulaziz Medical City, Jeddah, SAU

**Keywords:** brain malignancy, skull invasion, temporal bone invasion, brain tumor, tumor skull extension, gliosarcoma

## Abstract

Gliosarcoma (GS) is a primary central nervous system tumor. It is an unusual type of glioblastoma multiforme (GBM) and rarely invades the skull base. It has a biomorphic tissue pattern with rapid alternation zones of glial and mesenchymal differentiation. We report the case of a 62-year-old male who presented with a one-month history of unsteady gait associated with dizziness. Brain MRI showed a right temporal mass that invaded the skull base with perilesional edema and a significant mass effect on the right lateral ventricle. The patient underwent a right-sided frontotemporal craniotomy with gross total resection. The pathology confirmed the diagnosis of GS. Postoperatively, the patient had an uneventful recovery with no complications and was discharged two days post-surgery.

## Introduction

Gliosarcoma (GS) is a rare malignant type of glioblastoma multiforme (GBM) and accounts for 2% to 3% of all cases of GB [1]. The characteristic pathologic feature of GS is a biomorphic tissue pattern, with rapid alternation zones of glial and mesenchymal differentiation [2]. Its appearance is characterized by the rapid growth of intra-axial mass with heterogenous enhancement [3]. Gliosarcoma cases are known to have a bad prognosis with high prevalence in the 6th decade, and males are affected nearly twice in comparison to females. [4]. The average size of the tumor variant measured is about 4.5 cm [4]. Gliosarcoma is predominant in the temporal lobe of the brain and typically presents as a firm superficial lesion with meningeal adhesions [5]. The typical presentation of GS is after GBM excision and even after the reception of radiotherapy, and only a few cases reported GS with extracranial extension as primary presentation [4]. To our knowledge, we have reported the ninth case of primary intracranial GS with skull base invasion, and it is considered the first case reported in Saudi Arabia.

## Case presentation

History

A 62-year-old male patient was referred to the neurosurgery department because of a right temporal lobe ring-enhancing lesion on brain MRI. The patient was complaining of an unsteady gait that was associated with dizziness, increased by leaning forward, for one month. He had no headache, loss of consciousness, seizures, blurred vision, nausea, vomiting, or hearing or speech abnormalities. The patient has no history of malignancy or similar conditions in the family.

Physical examination

The patient was vitally stable with a 15/15 Glasgow coma scale (GCS). Pupils were 2 mm in diameter in bright light with normal eye movements and with no visual field defects. No facial muscle weakness, abnormal sensation, or tongue deviation were found. Also, the patient had normal muscle tone and power. He had normal sensations and reflexes in the upper and lower limbs.

Imaging

**Figure 1:**
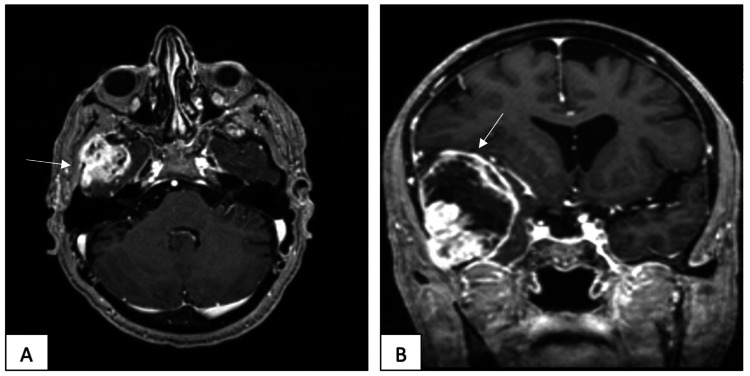
Preoperative MRI The axial (A) and coronal (B) views show a ring-enhancing lesion with central necrosis with midline shaft.

A gadolinium contrast brain MRI showed a 48 x 55 x 57 mm right temporal lobe mass with surrounding edema and a significant mass effect on the right lateral ventricle, midline shift, and right uncal herniation (Figure 1). Moreover, the characteristics of a mass were in keeping with high-grade glioma. Furthermore, abdominal US showed extensive liver cirrhosis with no focal hepatic lesions. 

Operation

Under general anesthesia in a supine position, the head was turned to the left, and the operative field was draped in the usual sterile fashion. A question mark incision was created, reflecting the scalp and the temporalis muscle. A fungating mass was found penetrating and eroding the temporal bone and attached to the temporalis muscle at the skull base. The tumor was mildly vascular with a firm consistency. Frontotemporal craniotomy was done dissecting around the fungating mass. The dura was invaded by the lesion, so the dura was opened in a curved linear fashion. A reasonable plane was created between the brain and the mass. A gross total resection of the temporal mass was made including the extracranial components, which was invading through the dura and the bone and its attachment to the temporalis muscle. The intraoperative frozen section confirmed the diagnosis of GS. Hemostasis was obtained, DuraSeal® was used to reconstruct the dura, the bone flap was secured with plates and screws, and the scalp flap was closed in layers. 

Histopathology

**Figure 2:**
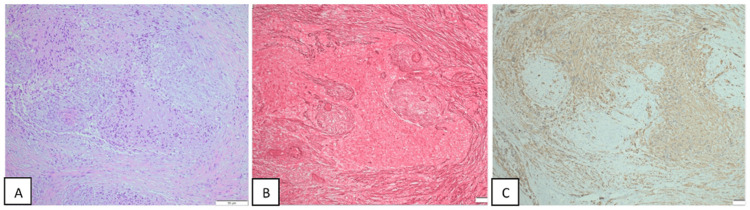
Immunohistochemistry H&E staining A: H&E stained sections show a biphasic mixture of gliomatous and sarcomatous tumors. The glial portion is astrocytic in nature with atypical nuclei and eosinophilic cytoplasmic processes. The sarcomatous portion is composed of densely packed long bundles of spindle cells. Mitotic figures, microvascular/endothelial proliferation, and areas of necrosis are seen. B: Reticulin shows a biphasic tissue pattern of reticulin-rich sarcomatous and reticulin-free gliomatous elements. C: Immunohistochemistry shows a GFAP-positive gliomatous component while being negative in the sarcomatous part. H&E: Hematoxylin & eosin; GFAP: Glial fibrillary acidic protein

The specimen illustrated a neoplasm invading dura. The tumor showed a biphasic mixture of gliomatous and sarcomatous tissue. The glial portion is astrocytic in nature with atypical nuclei and eosinophilic cytoplasmic processes. The sarcomatous portion was composed of densely packed long bundles of spindle cells. Mitotic figures, microvascular proliferation, and areas of necrosis were seen (Figure 2).

Postoperative follow-up

**Figure 3:**
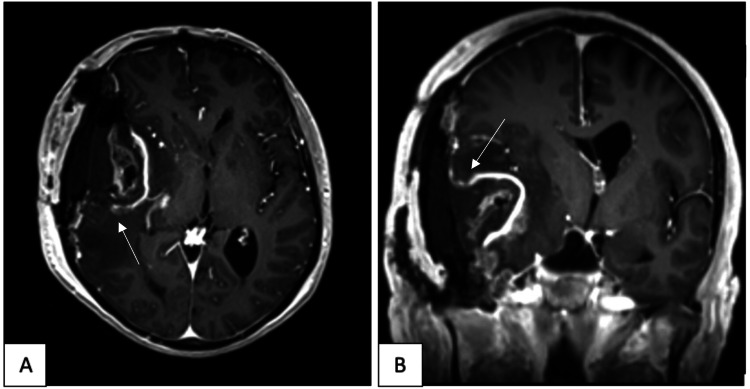
Postoperative T1-weighted gadolinium-enhanced MRI of the brain Axial (A) and coronal (B) views confirm subtotal resection of the temporal mass including the extracranial components.

**Figure 4:**
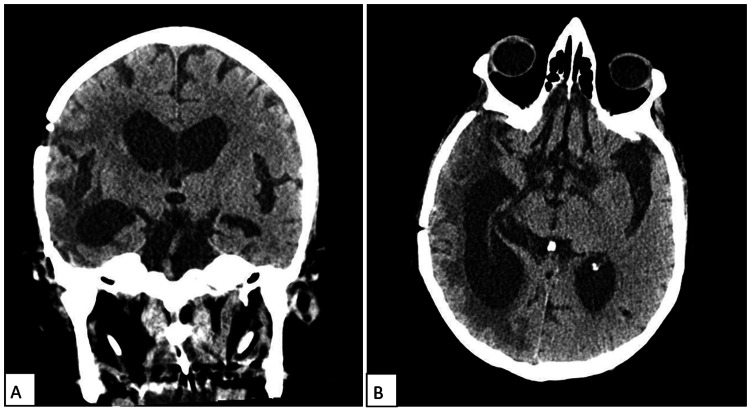
Postoperative CT The coronal (A) and axial (B) views show right pterional craniotomy and right cerebral hemispheric hypodensity with right lateral ventricle ex vacuo phenomenon.

The procedure was well tolerated. There was no complication, and the patient was sent to the intensive care unit (ICU) for observation and spent one day in ICU in stable condition. Postoperatively, the patient was conscious and oriented and had a GCS score of 15/15. Cranial nerves were intact without any neurological deficit. Postoperative MRI showed subtotal resection of the temporal mass from the right side and the extracranial components (Figures 3-4). On postoperative day 2, the patient was not in pain, orally well tolerating, and mobilizing out of bed. His condition was stable, so the patient was discharged on postoperative day 3. The patient was started on temozolomide as part of the management plan and followed up by the oncology department for further evaluation. 

## Discussion

Gliosarcoma is a rare malignancy of the brain that has a biomorphic tissue pattern including glial and mesenchymal differentiation. The exact prevalence of GS is not known. However, it has been reported to account for 2% of all GBM in adults, with a higher incidence in males [6]. Also, it has been reported to range from 1% to 8% of all gliomas [7]. In Saudi Arabia, this is the first reported case of skull base invasion. There are two components of GS: malignancy of the glial cell, or glioma; and sarcoma, which is histiocytoma in most cases [1,8]. In the absence of prior presentation with GBM, primary GS is an uncommon tumor that rarely extends beyond the dura. Furthermore, invasion of the skull base is considered extremely rare, in contrast to our case, where the primary presentation of the patient was aggressive as the tumor was invading through the dura and bone to reach the temporalis muscle [9]. A literature review of Pubmed, Central, and Google Scholar shows eight studies with GS with the invasion of the skull base or temporal bone (Table 1) [9-16].

**Table 1:** Review of GS cases with invasion of the skull bone in literature with an additional case M: Male; F: Female; N/A: Not Available; GS: Gliosarcoma

Authors	No. Of Patients	Age (in years)/Gender	Preoperative symptoms	Postoperative symptoms	First-time diagnosis with GS	Invasion	Complications
Nguyen et al. [9]	1	45 F	Difficulty opening the right jaw, swelling in the right cheek and temple region, right retro-orbital pressure, mild right proptosis, and right side paresthesias	N/A	At first presentation	Extension into the orbit, maxillary sinus, and invasion of the right pterygoid, masseter, and temporalis muscles	N/A
Maeda et al. [10]	1	51 F	Headache	Recurrent lesion in the intracranial temporal base after two months	At first presentation	Dura matter	died of respiratory failure 5 months after the initial diagnosis.
Oberndorfer et al. [11]	1	37 M	Headache, mild deficits of concentration, and mild mnestic dysfunction	Cluster headache-like syndrome on the right fronto-orbital facial region	Second presentation	Infiltration of the cavernous sinus, the skull base, and extension into cervical soft tissue, retro-orbital space	The patient died due to pneumonia 11 months after diagnosis. Postmortem investigation also revealed metastases to the diaphragm.
Sade et al. [12]	1	55 M	Headache and papilledema	N/A	First presentation	Extension into the infratemporal fossa and lateral sphenoid sinus	N/A
Schuss et al. [13]	1	54 M	Right facial pain along the maxillary branch of the trigeminal nerve for two months			Infiltrating the infratemporal fossa and the sphenoidal sinus, Meckel’s cave, foramen rotundum, and the meninges of the middle skull base on the right side	The patient died a few weeks later, 22 months after initial diagnosis.
Malde et al. [14]	1	13 F	Intermittent headache, vomiting, imbalance on walking, dimness of vision and impaired hearing on the left side of 2 weeks’ duration, truncal ataxia, bilateral horizontally evoked nystagmus, left-sided sensorineural deafness, and papilloedema	After eight years, intermittent holocranial headache, vomiting, giddiness, slurred speech, and early papilloedema.	Second presentation	N/A	Progressive worsening of symptoms and was put on palliative care
Nesvick et al. [15]	1	44 F	Difficulty opening the right jaw, retro-orbital pressure for three months, severe right-sided post-auricular headache for one week	N/A	First presentation	Extension through the right orbit and muscles of mastication	None
Liew et al. [16]	1	82 M	Gradual general weakness, poor response to speech for three weeks, poor awareness, and myoclonic jerks	N/A	First presentation	Extension into infratemporal fossa, via the foramen ovale, and to the wall of the temporal horn of the lateral ventricle, cavernous sinus, temporal bone, and sphenoid ridge	The patient died of pneumonia, and the survival time was 9.2 months.
Our case	1	62 M	Unsteady gait with the sensation of falling and dizziness for one month	N/A	Second presentation	Extension from the right temporal lobe mass and right uncal herniation	N/A

Regarding clinical presentations, headache is considered to be the most reported symptom, followed by unsteady gait [9-15,17]. On MRI, GS appears as an extra-axial lesion despite the intra-axial origin of the tumor, which in addition to being well-circumscribed with focal edema as well as a broad dural and dural base enhancement, can make it mimic meningioma [13]. Though the mechanism of GS extension is still unclear, Kawano et al. listed three possibilities: (I) perivascular or dural slits; (II) along the cranial or spinal nerves; or (III) through direct destruction of the cranial architecture. The genetic profile of GS is similar to GBM, except that there is no amplification or overexpression of the epidermal growth factor receptor [14].

The most common location to metastasize is the temporal lobe [12,13]. Other parts can be involved, such as the frontal lobe, ventricle, and cerebellum [8]. In addition, the invasion could also interfere with blood supply, which can lead to necrosis in some areas, such as the dura [14]. 

The management of GS includes surgery in addition to radiotherapy, chemotherapy, or chemoradiotherapy. However, a retrospective study of 75 patients concluded that radiotherapy is superior to temozolomide-based chemoradiation or adjuvant chemotherapy for improving the survival rate [6].

Several factors play a role in overall survival, including age at diagnosis, the extent of surgery, and adjuvant radiotherapy. Fifteen months is the average survival period if the patient is younger than 50 years old, while it would be seven months if the patient is older than 50 years old. Radiotherapy increases survival to 10 months [8]. 

## Conclusions

Gliosarcoma is a rare type of glioblastoma. We highlighted a patient with GS invading the temporal area with the skull base. Skull base involvement and how it affects the prognosis or survival of the patient are not yet known. However, we encourage an aggressive attempt at resection with proper skull base surgical technique to ensure optimal results. Therefore, conducting further research is important to have a better understanding of this condition.

## References

[1] Galanis E, Buckner JC, Dinapoli RP (1998). Clinical outcome of gliosarcoma compared with glioblastoma multiforme: North Central Cancer Treatment Group results. J Neurosurg.

[2] Louis DN, Perry A, Reifenberger G (2016). The 2016 World Health Organization Classification of Tumors of the Central Nervous System: a summary. Acta Neuropathol.

[3] Kozak KR, Mahadevan A, Moody JS (2009). Adult gliosarcoma: epidemiology, natural history, and factors associated with outcome. Neuro Oncol.

[4] Lutterbach J, Guttenberger R, Pagenstecher A (2001). Gliosarcoma: a clinical study. Radiother Oncol.

[5] Beaumont TL, Kupsky WJ, Barger GR, Sloan AE (2007). Gliosarcoma with multiple extracranial metastases: case report and review of the literature. J Neurooncol.

[6] Zaki MM, Mashouf LA, Woodward E (2021). Genomic landscape of gliosarcoma: distinguishing features and targetable alterations. Sci Rep.

[7] Meis JM, Martz KL, Nelson JS (1991). Mixed glioblastoma multiforme and sarcoma. A clinicopathologic study of 26 radiation therapy oncology group cases. Cancer.

[8] Borota OC, Scheie D, Bjerkhagen B, Jacobsen EA, Skullerud K (2006). Gliosarcoma with liposarcomatous component, bone infiltration and extracranial growth. Clin Neuropathol.

[9] Nguyen QD, Perry A, Graffeo CS (2016). Gliosarcoma with primary skull base invasion. Case Rep Radiol.

[10] Maeda D, Miyazawa T, Toyooka T, Shima K (2010). Temporal gliosarcoma with extraneural metastasis: case report. Neurol Med Chir (Tokyo).

[11] Oberndorfer S, Wöhrer A, Hainfellner JA, Calabek B, Tinchon A, Brandl I, Grisold W (2013). Secondary gliosarcoma with massive invasion of meninges, skull base, and soft tissue, and systemic metastasis. Clin Neuropathol.

[12] Sade B, Prayson RA, Lee JH (2006). Gliosarcoma with infratemporal fossa extension. J Neurosurg.

[13] Schuss P, Ulrich CT, Harter PN, Tews DS, Seifert V, Franz K (2011). Gliosarcoma with bone infiltration and extracranial growth: case report and review of literature. J Neurooncol.

[14] Malde R, Jalali R, Muzumdar D, Shet T, Kurkure P (2004). Gliosarcoma occurring 8 years after treatment for a medulloblastoma. Childs Nerv Syst.

[15] Nesvick CL, Perry A, Graffeo CS (2017). Primary intracranial gliosarcoma with extensive invasion of the skull base, brain parenchyma, orbit, and muscles of mastication. J Neurol Surg B Skull Base.

[16] Liew S-J, Liao W-J, Liu J-T, Tan W-C (2013). Gliosarcoma with extension to infratemporal fossa and ventricle. Formos J Surg.

[17] Murphy MN, Korkis JA, Robson FC, Sima AA (1985). Gliosarcoma with cranial penetration and extension to the maxillary sinus. J Otolaryngol.

